# Compassionate and respectful care among outpatient clients at public health facilities in Northwest Ethiopia: A mixed-methods study

**DOI:** 10.1371/journal.pone.0252444

**Published:** 2021-06-11

**Authors:** Manaye Abate, Ayal Debie, Chalie Tadie Tsehay, Tsegaw Amare

**Affiliations:** 1 Maternal and Child Health, Motta Health Office, Motta, Ethiopia; 2 Department of Health Systems and Policy, Institute of Public Health, College of Medicine and Health Sciences, University of Gondar, Gondar, Ethiopia; University of Mississippi Medical Center, UNITED STATES

## Abstract

**Introduction:**

Compassionate and respectful care is a pillar for improving health-seeking behaviour. It has given much attention globally, following the concerns that healthcare often falls significantly; however, less research emphasis was paid in the last decade. Therefore, this study aims to assess compassionate and respectful care and associated factors among outpatient clients at public health facilities in Northwest Ethiopia, 2020.

**Methods:**

A facility-based quantitative cross-sectional study supplemented with the qualitative findings was conducted from 30 February to 30 March 2020. A semi-structured interviewer-administered questionnaire was used to collect the quantitative data among 593 participants. Systematic and purposive sampling techniques were used to select the quantitative and qualitative participants, respectively. A semi-structured interview guide was also employed for the qualitative data collection. Epi-Data version 4.6 and SPSS version 25 were used for data entry and analysis, respectively. The bi-variable and multivariable logistic regression model was fitted to identify the factors associated with each outcome variable (compassionate and respectful care separately). Adjusted odds ratio (AOR) with 95% confidence interval (CI) and p-value less than 0.05 were used to declare the strength and factors statistically associated with the outcome variables.

**Results:**

Overall, 72.8% and 82.6% of the respondents experienced compassionate and respectful care, respectively. Patients attending primary or above education (AOR: 0.35; 95% CI: 0.21–0.59), attending at the hospital (AOR: 0.59; 95% CI: 0.39–0.88), new clients (AOR: 0.33; 95% CI: 0.16–0.70) and service users who had three or more visits (AOR: 0.34; 95% CI: 0.17–0.71) were negatively associated with compassionate care. On the other hand, female patients (AOR = 0.53; 95% CI: 0.32–0.87), aged over 36 years (AOR = 0.43; 95% CI: 0.20–0.90), primary or above school attended clients (AOR = 0.18; 95% CI: 0.09–0.36), waiting two or more hours (AOR = 0.28; 95% CI: 0.13–0.62), and use public or private transport access (AOR: 0.49; 95% CI: 0.29–0.83) were negatively associated with a respectful care.

**Conclusion:**

Compassionate and respectful care provided to the outpatient clients in public health facilities of northwest Ethiopia was high. However, our result suggests that immediate actions are necessary to address respectful and compassionate care at hospitals, and hospital management should adopt mitigation measures. Consideration should be given to foster patient-centric services and educate the health care workers about compassionate and respectful care.

## Introduction

The United Nations (UN) human rights declaration puts all human beings free and equal in dignity and rights [1]. Compassionate care is a feeling of deep kindness and grief for serving patients with an ethical and professional oath to alleviate their sufferings, whereas respectful care is care that supports and promotes patients’ self-respect regardless of any differences [2, 3]. Compassionate and respectful care within healthcare has given too much attention globally, following the concerns that healthcare often falls remarkably [4, 5]. However, less research emphasis was assumed on healthcare settings in the last decade [5].

Even though there was limited evidence in healthcare settings, a few available studies were done in the Czech Republic and Nigeria revealed that many women had practised disrespectful care during child delivery and, in turn, undermine health facilities’ level [6, 7]. On the other hand, the level of respectful maternity care in East and southern Africa was ranged from 62 to 67% [8].

Studies conducted in Ethiopia showed that an average number of service users (42.9 to 48.8%) had received both compassionate and respectful health care services [9–11], and only 59% of hospitals had delivered respectful care [12]. Moreover, workload and inadequate supplies were listed as the major contributors to low compassionate and respectful care [13–15].

Recognizing these gaps, the federal ministry of health (FMOH) Ethiopia incorporated compassionate and respectful care in the health sector transformation plan (HSTP) [4, 16]. Despite considering these healthcare services in the second growth and transformation plan, less emphasis was given on measuring its progress when HSTP is coming to an end. Therefore, this study aimed to assess the compassionate and respectful care and associated factors among outpatient clients at public health facilities of Hulet Eju Enesie district, Northwest Ethiopia.

The finding of this study will help the regional and national policy-makers and programmers to make evidence-based decision to enhance the health systems responsiveness and to increase the community health-seeking behaviour.

## Materials and methods

### Study design and settings

A facility-based cross-sectional quantitative study supplemented with the qualitative inquiry was conducted from 30 February– 30 March 2020 among outpatient clients at public health facilities in Hulet Eju Enesie district, Northwest Ethiopia. The quantitative study aimed to determine the level of compassionate and respectful care among outpatient clients. The sequential explanatory design was employed to explain and interpret quantitative results by collecting and analyzing, followed by qualitative data to explore the key factors that enable and deters compassionate and respectful care in the study area.

Hulet Eju Enesie district is one of the East Gojjam zone districts of Amhara National Regional State, located 371 and 120 km away from Addis Ababa (capital city of Ethiopia) and Bahir Dar (the capital city of Amhara National Regional State), respectively. The district has 28 kebeles (the lowest administrative unit in Ethiopia) with a predicted total population of 336,465 with predominantly rural (283,077) residents in 2017 [17]. The district has nine health centres and one primary hospital. The average number of patients attending the outpatient department (OPD) per month at the hospital and health centres was 5,200 and 1,250 clients.

### Population

All patients who had received healthcare services at the OPD of public health facilities in Hulet Eju Enesie district were the source population. Those patients who attended the OPD in the selected public health facilities were the study population. Unit heads, OPD coordinators, and outpatient clients were recruited for the qualitative inquires. Patients who could not communicate, below 18 years old, had two or more health facility visit during the data collection period, and critically ill patients were excluded from the study.

### Sample size determination

The sample size was calculated using a single population proportion formula considering statistical assumptions, the proportion of compassionate care in Tikur Anbesa hospital (45.7%) [10], respectful care in Addis Ababa (50.3%) [18], 5% margin of error, 10% non-response rate and 1.5 design effect. A total of 629 and 634 samples were used to assess the level of respectful and compassionate care, respectively. However, 634 was taken as the final sample size to maintain the adequacy of the sample sizes.

A total of eight participants (both in-depth and key informant) interviews had involved in the qualitative study. Five key informant interviews (KIIs) (one health centre head and two case team coordinators at the health centre and one unit head and one case team coordinator at the hospital) and three in-depth interviews from patients were conducted. The sample size for the qualitative study was determined based on saturation of information.

### Sampling procedures

A multi-stage random sampling followed by a stratified sampling technique was employed to select quantitative data collection participants. Three health centres were selected randomly, and a hospital was chosen purposely, and samples were proportionally allocated based on the total OPD patients. Finally, the participants were selected using a systematic sampling technique. A purposive sampling technique was used to select the participants for qualitative inquiry. Participants were chosen according to their ease of access, exposure, and experience on compassionate and respectful care.

### Study variables and measurements

Compassionate and respectful care were the dependent variables of the study. The independent variables include age, sex, religion, marital status, educational status, residence, occupation, income, type of OPD, type of health facility, number of visits, waiting time, distance to the health facility, mode of transport and source of information. Compassionate care was measured by using 15 item questions; each contains a five-point Likert scale. A patient who scored above 60% from the total compassionate care measurement scores was considered as receiving good compassionate care, and the remaining was deemed to receive poor compassionate care.

Respectful care was measured by using 16 items questions; each contains a five-point Likert scale. A patient who scored above 60% from the total respectful measurement score was considered to receive as good respectful care, and the remaining was considered as getting poor respectful care.

### Data collection tools and procedures

A semi-structured interviewer administered questionnaire was developed by reviewing various literature [9–12] and mainly adapted from the compassionate and respectful care module prepared by the FMOH of Ethiopia [4] to measure the quantitative variables. The questionnaire includes respondents’ sociodemographic characteristics, institution related factors, source of information related items, and compassionate and respectful care measurement items. An interview guide was also prepared by reviewing different articles by the principal investigator [13–15] to explore the cares’ enablers and barriers. An in-depth interview for the patients and key informant interviews for the KIIs were conducted by maintaining their privacy and mobile recorder to record the interview process.

### Data quality assurance

The questionnaire was prepared in English, then translated to Amharic (local language) and back to English by language experts to check the consistency. A pretest was conducted on 32 OPD patients at Kernewary and Sedie health centres. Necessary modifications were made based on the pretest findings to improve the clarity, understandability, and simplicity of the messages. A day of training was given for data collectors and supervisors on the study’s objectives, benefits, individual’s right to accept or refuse the interview, informed consent, and techniques of the interview. Regular meetings were held between the data collectors, supervisors, and principal investigators throughout the data collection period. Any problems or challenges faced at the time of the data collection process were discussed, and corrective measures were taken. The collected data were again reviewed and checked for completeness before data entry. A seminar presentation was held for the study population (mainly for professionals) to check the qualitative information’s truthfulness.

### Data processing and analysis

Data were entered into Epi data version 4.6 and exported to SPSS version 25 software for analysis. Descriptive statistics, such as mean and frequency, were presented using texts and tables. Binary logistic regression model fitted to identify the independent variables with the outcome variables. Independent variables with a p-value of less than 0.2 during binary logistic regression analysis were eligible for the multivariable logistic regression model. Variables that failed to attain the chi-square and multicollinearity assumptions were removed from multiple logistic regression analysis. The presence of multicollinearity was examined using the Variance Inflation Factor (VIF), and a variable having a VIF value (>10) was rejected. Adjusted Odds Ratio (AOR) with 95% CI and p-value less than 0.05 were used to declare the strengths and the factors significantly associated with each outcome variable (compassionate and respectful care separately). The mobile-recorded qualitative data were transcribed and translated to English for analysis. The qualitative data were changed into plain text and analyzed using thematic analysis using Open code software.

### Ethical consideration

Ethical clearance was obtained from the ethical review committee of the Institute of Public Health of College of Medicine and Health Sciences at the University of Gondar with a reference number of IPH/837/6/2012. A supportive letter was taken for each health facility from Amhara Public Health Institute. Each participant was assured of the confidentiality of the information they provide. Written informed consent for quantitative study participants and oral informed consent for qualitative study participants were obtained.

## Results

### Sociodemographic characteristics of respondents

A total of 593 patients participated in the study, with a response rate of 93.5%. Nearly one third (30.5%) of participants were aged 26–35 years with the mean age of 37.8 (±SD of 13.3) years and median of 36 (± IQR of 19) years. More than half (53.6%) of the participants were male, and 39.1% were unable to read and write. Nearly ninety per cent (86.5%) were Orthodox Christians, and two-thirds (68.8%) were married. Moreover, over three-quarter (79.3%) of the participants lived in rural areas (Table 1).

**Table 1:** Sociodemographic characteristics of respondents among outpatient clients at public health facilities, Northwest Ethiopia, 2020 (n = 593).

Variables	Category	Frequency	Percent (%)
Age in years	18–25	114	19.2
	26–35	181	30.5
	36–45	146	24.6
	46–55	75	12.6
	>55	77	13.0
Sex	Male	318	53.6
	Female	275	46.4
Religion	Orthodox	513	86.5
	Muslim	76	12.8
	Others*	4	0.7
Marital status	Married	408	68.8
	Single	121	20.4
	Divorced	27	4.6
	Widowed	27	4.6
	Separated	10	1.7
Occupation	Farmer	279	47.0
	Civil servant	63	10.6
	Self-employed	110	18.5
	Housewives	82	13.8
	Students	59	9.9
Educational status	Cannot read and write	232	39.1
	Can read and write	176	29.7
	Primary school and above	185	31.2
Residence	Rural	470	79.3
	Urban	123	20.7
Average household income (ETB)	Less than 2000	215	36.3
	2001–3000	146	24.6
	3001–4000	120	20.2
	More than 4000	112	18.9

*Catholic and protestants; ETB: Ethiopian Birr.

On the other hand, six health professionals and five patients for KIIs and in-depth interviews participated in the qualitative part, respectively. Half of the KIIs were taken from health centres, and three patients were also involved in the in-depth interview from health centres.

### Institution related characteristics of the participants

Two-thirds (66%) of the participants were taken from health centres, and one-fifth of the total patients (19.6%) involved in the study were attending an emergency OPD. The average waiting time to receive services for almost half of the participants (49.9%) was 2.45 hours. About two-thirds (63.6%) and only 10.3% had got laboratory and radiology services, respectively. Moreover, the majority (94.3%) of the participants had received a pharmacy service (Table 2).

**Table 2:** Institution-related characteristics of participants among outpatient clients at public health facilities, Northwest Ethiopia, 2020 (n = 593).

Variables	Category	Frequency	Percent (%)
Type of health facility	Health centre	397	66.0
	Hospital	196	34.0
Type of OPD	Emergency	116	19.6
	Cold case	477	80.4
Type of service	New	472	79.6
	Repeated/follow up	121	20.4
Number of visits for the last six months	No	332	56.0
	One	91	15.3
	Two	65	11.0
	Three or more	105	17.7
Waiting time	Less than two hours	97	16.4
	Two hours	296	49.9
	More than two hours	200	33.7
Laboratory service	No	216	36.4
	Yes	377	63.6
Radiology service	No	532	89.7
	Yes	61	10.3
Pharmacy service	No	34	5.7
	Yes	559	94.3
Counselling and others	No	581	98.0
	Yes	12	2.0
Number of service units visited	Three	64	10.8
	Four	183	30.9
	Five	261	44.0
	Six or more	85	14.3

### Physical and information access to the respondents

More than sixty per cent (63.6%) of the respondents lived ten or more km away from the health facility, and again nearly sixty per cent (58.2%) of the participants had gone to the health facility on foot. On the other note, only 37.8% of the clients had information about compassionate and respectful care. On top of that, only one third (34.7%) of those respondents had got information from television, followed by formal education (30.9%) (Table 3).

**Table 3:** Information and physical-related characteristics of respondents among outpatient clients at public health facilities, Northwest Ethiopia, 2020 (n = 593).

Variables	Category	Frequency	Percent (%)
Distance to the nearest health facility (km)	<10	216	36.4
	10–30	271	45.7
	>30	106	17.9
Mode of transport	Foot	224	37.8
	Public or private	369	62.2
Ever heard of compassionate and respectful care	No	329	55.5
	Yes	264	44.5
Source of information	Television	92	34.7
	Radio	79	29.8
	Formal education	82	30.9
	Others*	12	4.5

*Friends, families and colleagues.

#### Compassionate care

Overall, 72.8% (95% CI: 69.3% - 76.4%) of respondents had received a good compassionate care. The majority of the respondents (nearly 70.0%) had got poor greetings during the entrance and dissatisfied with the release of bad breaking news by the healthcare providers in the presence of their family. On the other hand, more than eighty-five per cent (86.2%) of clients reported that providers actively listened to their concerns and 79.8% of patients also replied that providers were properly understood their need and showed love and tolerance for them during the service delivery (Table 4).

**Table 4:** Level of compassionate care among outpatient clients at public health facilities, Northwest Ethiopia, 2020 (n = 593).

Items	SD, n (%)	D, n (%)	N, n (%)	A, n (%)	SA, n (%)	Total	
						Poor (%)	Good (%)
**During entrance and greeting**							
The Provider properly introduces himself/herself	104 (17.5)	301 (50.8)	4 (0.7)	157 (26.5)	27 (4.6)	409 (69.0)	184 (31.0)
The Provider introduced her/his name	91 (15.3)	343 (57.8)	3 (0.5)	128 (21.6)	28 (4.7)	437 (73.7)	156 (26.3)
**During procedure**							
The Provider engage himself/herself with the you	8 (1.3)	130 (21.9)	7 (1.2)	400 (67.5)	48 (8.1)	145 (24.5)	448 (75.5)
The Provider actively listened what you are said	9 (1.5)	69 (11.6)	4 (0.7)	420 (70.8)	91 (15.3)	82 (13.8)	511 (86.2)
The Provider showed love and tolerance	5 (0.8)	113 (19.1)	2 (0.3)	411 (69.3)	62 (10.5)	120 (20.2)	473 (79.8)
The Provider tried to understand your need	7 (1.2)	111 (18.7)	2 (0.3)	389 (65.6)	84 (14.2)	120 (20.2)	473 (79.8)
The Provider show relational communication	5 (0.8)	135 (22.8)	3 (0.5)	383 (64.6)	67 (11.3)	143 (24.1)	450 (75.9)
The Provider use supportive words	5 (0.8)	205 (34.6)	8 (1.3)	331 (55.8)	44 (7.4)	218 (36.8)	375 (63.2)
The Provider responds promptly and professionally	3 (0.5)	130 (21.9)	3 (0.5)	381 (64.2)	76 (12.8)	136 (22.9)	457 (77.1)
The Provider tried to address your spiritual need	4 (0.7)	188 (31.7)	6 (1.0)	340 (57.3)	55 (9.3)	198 (33.4)	395 (66.6)
The Provider tried to involve you in treatment options	11 (1.9)	164 (27.7)	5 (0.8)	342 (57.7)	71 (12.0)	180 (30.4)	413 (69.6)
The Provider frequently communicates the healthcare team regarding your treatment	7 (1.2)	154 (26.0)	7 (1.2)	362 (61.0)	63 (10.6)	168 (28.3)	425 (71.7)
The Provider breaks the bad news in the presence of their family	75 (12.6)	271 (45.7)	68 (11.5)	168 (28.3)	11 (1.9)	414 (69.8)	179 (30.2)
The Provider spends enough time with you	5 (0.8)	151 (25.5)	2 (0.3)	412 (69.5)	23 (3.9)	158 (26.6)	435 (73.4)
The Provider strives to understand your emotional needs	16 (2.7)	221 (37.3)	12 (2.0)	307 (51.8)	37 (6.2)	249 (42.0)	344 (58.0)

SD: Strongly Disagree; D: Disagree; N: Neutral; A: Agree; SA: Strongly Agree.

### Respectful care

In this study, 82.6% (95% CI: 79.57% - 85.26%) of clients were received substantial respect during healthcare. Two thirds (68.3%) of patients were dissatisfied with the level of respectful care related to provider self-introduction during the entrance time, and 71.2% of respondents were handled disrespectfully at the time of receiving the provider’s contact address after completion of the service delivery. Most patients (87.4%) reported that the healthcare providers actively listened to their cases, and providers assured 85.2% of the clients of the confidentiality of their information. The majority of the participants (80.4%) had received good respectful care during the procedure compared with the entrance time (Table 5).

**Table 5:** Level of respectful care among outpatient clients at public health facilities, Northwest Ethiopia, 2020 (n = 539).

Items	SD, n (%)	D, n (%)	N, n (%)	A, n (%)	SA, n (%)	Respectful care	
						Poor (%)	Good (%)
**During entrance and readiness**							
The Provider greets me respectfully	48 (8.1)	115(19.4)	1 (0.2)	328(55.3)	101 (17.0)	164 (27.7)	429 (72.3)
The Provider introduces him/herself	104(17.5)	298(50.3)	3 (0.5)	147(24.8)	41 (6.9)	405 (68.3)	188 (31.7)
The Provider properly addresses considering my social status	5 (0.8)	91 (15.3)	1 (0.2)	347(58.5)	149 (25.1)	97 (16.4)	496 (83.6)
**Care during procedure**							
The Provider actively listens to me	4 (0.7)	68 (11.5)	3 (0.5)	426(71.1)	92 (15.5)	75 (12.6)	518 (87.4)
The Provider allocates adequate time to talk to me	7 (1.2)	169(28.5)	6 (1.0)	363(61.2)	48 (8.1)	182 (30.7)	411 (69.3)
The Provider respects my view on treatment and care	6 (1.0)	138(23.3)	3 (0.5)	379(63.9)	67 (11.3)	147 (24.8)	446 (75.2)
The Provider obtain consent before any procedures	6 (1.0)	159(26.8)	2 (0.3)	349(58.9)	77 (13.0)	167 (28.2)	426 (71.8)
The Provider ensures confidentiality of my information	2 (0.3)	85 (14.3)	1 (0.2)	423(71.3)	82 (13.8)	88 (14.8)	505 (85.2)
The Provider maintains my privacy in providing clinical care	3 (0.5)	126(21.2)	8 (1.3)	377(63.6)	79 (13.3)	137 (23.1)	456 (76.9)
The Provider has good communication within the team	4 (0.7)	96 (16.2)	5 (0.8)	404(68.1)	84 (14.2)	105 (17.7)	488 (82.3)
The Provider treats us equally without discrimination	4 (0.7)	100(16.9)	0 (0)	402(67.8)	87 (14.7)	104 (17.5)	489 (82.5)
The Provider responds promptly when I asked for help	6 (1.0)	114(19.2)	5 (0.8)	378(63.7)	90 (15.2)	125 (21.1)	468 (78.9)
**Communication after procedure**							
The Provider gives information about my treatment and care	6 (1.0)	104(17.5)	3 (0.5)	415(70.0)	65(11.0)	113 (19.1)	480 (80.9)
The Provider confirms my understanding with respect	5 (0.8)	167(28.2)	3 (0.5)	362 (61.0)	56 (9.4)	175 (29.5)	418 (70.5)
The Provider provides when to return with at most respect	4 (0.7)	100(16.9)	100(16.9)	328(55.3)	61 (10.3)	204 (34.4)	389 (65.6)
He/she provides contact address to those who need it	8 (1.3)	127(21.4)	287(48.4)	150 (25.3)	21 (3.5)	422 (71.2)	171 (28.8)

SD: Strongly Disagree; D: Disagree; N: Neutral; A: Agree; SA: Strongly Agree.

### Factors associated with compassionate care

Clients with primary or above education were less likely to receive compassionate care by 65% (AOR: 0.35; 95% CI: 0.21–0.59) than those who did not read and write. A case team coordinator at one of the health centres replied that "*compassionate care practice is quite different and varies from professional to professional both in type and extent*. *I think personal characteristics matter more than the educational level and the providers’ academic performance*. *Even it could have no value for their compassionate care practices*. *He also says that the healthcare providers are focusing on providing technical/clinical services to users even if some are trying their best to provide more compassionate care*"(a 22 years old case team coordinator at the health centre).

Patients attending the hospital were less likely to get compassionate care by 41% (AOR: 0.59; 95% CI: 0.39–0.88) compared with patients attending health centre. The qualitative study supported this finding. In the qualitative inquiry, the major contributors to poor compassionate care were the professionals’ high workload. Almost all professionals served beyond their capacity to deliver. These made them tired and bored, and affect to provide compassionate care. The KIIs and the in-depth interview participants indicated that the healthcare providers compassionate care practice was not that satisfactory, and lesser practice was observed in the hospital than in the health centres. The case team coordinator from the hospital replied, *"Even though the hospital is a primary hospital*, *the patient flow is too much and looks like a tertiary hospital*. *As a result*, *the number of health professionals and patient flow is not proportional*. *The case flow is much high compared with the standards*. *One professional can serve too many patients per day*, *particularly in the laboratory and pharmacy departments*. *This might lead to deliver poor compassionate care"* (a 32 years old male case team coordinator from the hospital).

Moreover, new clients were less likely to receive compassionate care by 67% (AOR: 0.33; 95% CI: 0.16–0.70) than patients who had received a follow care. On top of that, the odds of OPD service users who had three or more visits in the last six months were less likely to get compassionate care by 66% (AOR: 0.34; 95% CI: 0.17–0.71) compared with its counterparts (Table 6).

**Table 6:** Logistic regression analysis to identify factors statistically associated with compassionate care at public health facilities, Northwest Ethiopia, 2020 (n = 593).

Variables	Category	Compassionate care		COR (95% CI)	AOR (95% CI)
		Poor	Good		
Age in years	18–25	36	78	1	1
	26–35	51	130	1.18(0.71–1.96)	1.07(0.62–1.84)
	36 or more	74	224	1.40(0.87–2.25)	0.93(0.53–1.64)
Educational status	Unable to read and write	47	185	1	1
	Able to read and write	44	132	0.76(0.48–1.22)	0.64(0.39–1.05)
	Primary school or above	70	115	0.42(0.27–0.65)	0.35(0.21–0.59) *
Type of institution	Health center	95	302	1	1
	Hospital	66	130	0.62(0.43–0.90)	0.59(0.39–0.88) *
Type of service	New	134	338	1.38 (1.06–2.21)	0.33(0.16–0.70) *
	Follow up	27	94	1	1
No of visits in the last six months	No	100	232	1	1
	One	16	75	2.02(1.12–3.64)	1.94(1.06–3.55) *
	Two	10	55	2.37(1.16–4.84)	2.08(1.00–4.33)
	Three or more	35	70	0.86 (0.54–1.38)	0.34(0.17–0.71) *
Mode of transport	Foot	65	159	1	1
	Public(private) transport	96	273	1.16(0.83–1.68)	1.17(0.79–1.75)

*Significant at p<0.05, COR: Crud Odds Ratio, AOR: Adjusted Odds Ratio, CI: Confidence Interval.

In the qualitative findings, KII participant also reported that many health professionals have good knowledge about compassionate care in hospital, but occasionally awareness gap was observed among some health professionals. A case team coordinator at the hospital replied, *"There are professionals who had poor knowledge about how we can handle our patients compassionately* " (a 32 years old male case team coordinator from the hospital). On top of that, an educated male patient at the health centre reported "*sometimes it seems that some health professionals did not learn about health or medical ethics because they did not show any compassionate care for their clients and I perceived that they might not have good knowledge about it*" (35 years old educated male patient from the health centre).

Case team coordinators at the health centre and the hospital also reported that *"some professionals have a poor attitude towards compassionate care*. *This could be due to some health care providers may not happy with their profession and the payment they got on that profession*. *This affects not only compassionate care delivery but also the overall hospital services performances*.*"* (a 28 and 32 years old male case team coordinators at the health centre and hospital).

One of the other barriers to compassionate care is low community acceptance towards health professionals. This affects the attitude of professionals and directly or indirectly affects compassionate care practices. A health centre head reported that *"Before few years*, *health professionals had good acceptance by the community members*, *which enhanced their work performance*, *but nowadays*, *acceptance of health professionals is becoming low even near zero*. *This will damage their motives towards their job and*, *in turn*, *affects the provision of compassionate care"* (a 24 years old male head of the health centre).

### Factors associated with respectful care

Female patients were less likely to receive respectful healthcare services by 47% (AOR = 0.53; 95%CI: 0.32–0.87) compared to their counterparts. Patients aged over 36 years were less likely to receive respectful healthcare by 57% (AOR = 0.43; 95%CI:0.20–0.90) than early adult (18–25) patients. Primary or above school attended clients were less likely to have received respectful care than those unable to read and write by 82% (AOR = 0.18; 95%CI:0.09–0.36). Patients waiting two or more hours at the health facility were 72% (AOR = 0.28; 95%CI: 0.13–0.62) less likely to get respectful care than patients waiting for below two hours. Patients who had public or private transport access were less likely to get respectful care by 51% (AOR: 0.49; 95% CI: 0.29–0.83) than patients who came on foot. Patients who had received radiology service were less likely to receive respectful care by 69% (AOR = 0.31; 95%CI: 0.16–0.60) compared with their counterparts (Table 7).

**Table 7:** Logistic regression analysis to identify factors associated with respectful care at public health facilities, Northwest Ethiopia, 2020 (n = 593).

Variables	Category	Respectful care		COR (95% CI)	AOR (95% CI)
		Poor	Good		
Age in years	18–25	20	94	1	1
	26–35	37	144	0.83(0.46–1.51)	0.59(0.30–1.17)
	36 or more	46	252	1.17(0.66–2.07)	0.43(0.20–0.90) *
Sex	Male	43	275	1	1
	Female	60	215	1.79 (1.16–2.75)	0.53(0.32–0.87) *
Educational status	Unable to read and write	20	212	1	1
	Able to read and write	38	138	3.41(1.93–6.02)	0.27(0.14–0.51) *
	Primary or above	45	140	1.17(0.71–1.91)	0.18(0.09–0.36) *
No of visits in the last six months	No	65	267	1	1
	One	7	84	1.15(0.67–1.97)	3.22(1.37–7.58) *
	Two	8	57	3.37 (1.37–8.27)	1.49(0.63–3.50)
	Three or more	23	82	2.00(0.84–4.78)	0.63(0.34–1.16)
Waiting time	Less than 2 hours	9	88	1	1
	2 hours	37	259	3.90(1.84–6.26)	0.67(0.30–1.49)
	More than 2 hours	57	143	2.79(1.76–4.43)	0.28(0.13–0.62) *
Radiology service	No	78	454	1	1
	Yes	25	36	4.04(2.30–7.10)	0.31(0.16–0.60) *
Mode of transport	Foot	29	195	1	1
	Public (private) transport	74	295	0.59(37–0.95)	0.49(0.29–0.83) *

*Significant at p<0.05, COR: Crud Odds Ratio, AOR: Adjusted Odds Ratio, CI: Confidence Interval.

The KIIs reports showed that respectful healthcare was not exercised at the health facility with its expected level even though some healthcare providers were trying to practice its components. They also replied that its practice was much lesser in hospital than in health centres. The KII participant reported that "*professionals in hospital practice many disrespectful actions*, *but this does not mean that all of their practices are disrespectful*. *Healthcare professionals are trying to give respectful and compassionate care*, *but it is found to be below the expected level*" (a 38 years male respondent from one of the hospital units).

On the other hand, an in-depth interview with patient indicated that most of the respondents experienced disrespectful care at least once in their stay at the health facility. The respondents claim supportive staffs are more disrespectful than healthcare professionals. An in-depth interview with patients from the hospital reported, "*There are many professionals who gave service respectfully we have to give credit to them*, *but some providers are even insulting patients and their attendants*. *On the other hand*, *I am really grateful for all professionals when I saw supportive staffs*, *especially working at a card room in almost all health facilities and the services delivered from these units are full of disrespects*"(a 32 years old male patient who had followed-up care for the last three years in the hospital).

Another in-depth interviewed patient also reported, "*I can say that I am a customer for this facility*. *I came to this health facility too many times for myself and families*. *I observed that there were too many disrespectful activities on myself and my other colleagues or clients*. *Even I observed that a patient who came with community-based health insurance was not considered a sick patient and did not get appropriate services*. *They were much more treated these patients disrespectfully*." (a 25 years old patient attending the health centre).

A KII from the hospital stated, "*Most health professionals have good knowledge about respectful care*, *and I don’t think that there is a health professional who did not know about respectful care*, *but they are practising in the wrong way*. " (a 32 years old male case team coordinator from the hospital).

## Discussion

This study investigated compassionate and respectful care provision in outpatient departments of public health facilities in Hulet Eju Enesie district. The study revealed that 72.8% (95% CI: 69.3% - 76.4%) of outpatient clients experienced good compassionate care at the public health facilities. The result is higher than a study in Tigray Ethiopia (55%) [19]. The variation might be due to the difference in time, study design, study participants and measurement tools. For instance, Tigray’s study used a single measurement tool to measure both compassionate and respectful care as a single outcome variable. On the other hand, the variation might indicate improved health care delivery throughout the country. The KII and patients’ in-depth interviews indicated that the health professionals’ practice and patients’ compassionate care experience were unsatisfactory. The finding contradicted a study conducted in Addis Ababa on oncology patients, as compassionate care was satisfactory for most respondents [10]. The discrepancy might be due to the difference in study settings that the later study was conducted on patients in the tertiary hospital where many interventions first applied at the national level.

The study also revealed that 82.6% (95% CI: 79.6–85.3) of clients visiting the outpatient department experienced good respectful care. This finding was also relatively higher than the studies among public hospitals at the national level in Ethiopia (5%) [12] and in Tigray, Ethiopia (55%) [19]. This discrepancy may also be due to the differences in study settings and period. The discrepancy also implies no uniformity in the implementation of respect full care across the country, which could have been improved by on job training and supportive supervision. However, this finding is lower than the study in east and south African countries [8], where 82.9% of patients experienced respectful care. It might be explained by differences in patient load and socio-cultural differences in patients’ perception of respectful care among the countries.

The KIIs finding supports the finding that health professionals practised compassionate and respectful care below the expected level and much less practised in hospital than health centres. This finding is in line with studies conducted in other parts of Ethiopia; Benishangul Gumuz [13], Tigray [14, 19] and Oromia [15], where implementation of respectful care was below the expected level. It related to high patient flow at the hospital; in the Ethiopian context, most of the community wants to be treated in hospitals than health centres regardless of the severity of the problem even for intestinal parasites and minor infections like common cold causes the unnecessary burden for the hospital. Because of professional’s burnout, the likely hood of poor compassionate and respectful caring is high. The rationale is in line with the qualitative finding that workload is one barrier for the less practice of compassionate care.

Besides, the study revealed that study participants who came for the first time into health facilities in the last six months were more likely to get respectful care than those who had a follow-up. This finding was in line with the study in Bahirdar [20]. It might be related to exhaustion due to repeated visits without progress or unnecessary appointment, direct and indirect cost of treatment, and inability to get seniors.

The study also indicated that aged patients were less likely to receive respectful care than young adults. This finding was supported by the studies done in Bahirdar [20] and Tigray [19]. This is because of the existence of some unethical professionals that contradicted Ethiopians’ culture. In Ethiopia, aged people are more respected in health care facilities and every aspect. This can be taken as the deviation of the new generation from the custom and beliefs of Ethiopia. However, the finding is contrary to the studies conducted in the Czech Republic [6] and Nigeria [7].

The study also indicated that primary or above-educated clients were less likely to get good compassionate, and respectful care than those who cannot read and write. It was supported by research conducted in Bahirdar [20]. This might be due to the complex raised due to peer education between the clients and the health care provider.

In this study, patients waiting for more than two hours were less likely to get compassionate care than patients waiting for less than two hours. This might be due to the high psychological impact of long waiting leading to a rough relation between the health providers and the clients [21]. On top of this, patients who came by public transport were more likely to get good compassionate care than patients who came to OPD by foot. This might be related to the fatigue raised due to the transport leads to communicate the health providers impatiently.

The key informant interviews of professionals and in-depth interview of patients revealed the barriers to implementing compassionate and respectful care at the public health facilities. The identified barriers were the knowledge gap of health professionals on compassionate and respectful care, low health professionals’ attitude, decreased community acceptance of health professionals, work overload and community illiteracy. This was in line with a qualitative study in Tigray [14] but contrary to a study conducted in Addis Ababa [10] on oncology patients that revealed a shortage of beds and different physicians treated as compassionate care barriers.

The application of mixed-method design can be the strength of this study. However, this study has limitations that should be considered while interpreting and concluding its result. The study’s finding might be prone to a response bias due to the patients’ self-report, and it does not provide an objective measure of compassionate and respectful care. The study’s cross-sectional nature doesn’t lead to making the causality of factors to the outcome variable.

## Conclusion

The overall compassionate and respectful care in the outpatient departments were high. However, our result suggests that immediate actions are necessary to address respectful and compassionate care at hospitals, and hospital management should adopt mitigation measures. Consideration should be given to foster patient-centric services and educate the health care workers and supportive staffs about compassionate and respectful care. Reducing facilities workload and improving community awareness on healthcare delivery also contributes to providing respectful and compassionate care. The next researcher shall conduct further institution-based research using an observational checklist.

## Supporting information

S1 Data (SAV)
